# Albumin Reduces Hepatic Steatosis and Inflammation in High-Fat-Diet-Fed Mice

**DOI:** 10.3390/ijms26157156

**Published:** 2025-07-24

**Authors:** Claire Rennie, Sheila Donnelly, Kristine McGrath

**Affiliations:** 1 School of Life Sciences, University of Technology Sydney, Sydney, NSW 2007, Australia; claire.rennie@uts.edu.au (C.R.); sheila.donnelly@universityofgalway.ie (S.D.); 2 Australian Institute for Microbiology and Infection, University of Technology Sydney, Sydney, NSW 2007, Australia; 3 School of Biological and Chemical Sciences, University of Galway, H91 TK33 Galway, Ireland

**Keywords:** MASLD, albumin, steatosis, high-fat diet, inflammation, mice

## Abstract

There are currently no approved therapeutic treatments targeting metabolic dysfunction-associated steatotic liver disease (MASLD). Albumin, a liver-produced plasma protein with anti-inflammatory and antioxidant properties, is reduced in advanced liver disease. Considering the role of chronic obesity-induced inflammation in MASLD pathogenesis, we investigated whether albumin administration could prevent disease progression to metabolic dysfunction-associated steatohepatitis (MASH). MASLD was induced in mice using a high-fat and high-cholesterol (PC) treatment for 8 weeks, followed by treatment with bovine serum albumin (BSA; 0.8 mg/kg) every three days for another 8 weeks. This regimen prevented time-dependent weight gain, regardless of diet, with 57% and 27% reductions in mice fed a standard chow (Std Chow) or PC diet, respectively. Further, supplementation reduced nuclear factor kappa B (NF-κB) activation by 2.8-fold (*p* = 0.0328) in PC-fed mice, consistent with albumin’s known anti-inflammatory properties. Unexpectedly, albumin also reduced hepatic neutral lipid accumulation and circulating non-esterified fatty acids. While PC-fed mice did not exhibit full progression to MASH, albumin treatment significantly increased hepatic matrix metalloproteinase-2 expression, suggesting the inhibition of early fibrotic signalling. While further studies are needed to elucidate the underlying mechanisms, these findings offer new insight into the potential of albumin, either alone or in combination with other therapies, to reduce hepatic steatosis in MASLD.

## 1. Introduction

Metabolic dysfunction-associated steatotic liver disease (MASLD), formerly non-alcoholic fatty liver disease, is a broad term that refers to liver conditions associated with excess fat accumulation in the liver (steatosis) in the context of metabolic dysfunction. It falls under the umbrella of steatotic liver disease (SLD), which refers to hepatic steatosis in the absence of significant alcohol consumption. The simplest and most benign form of SLD is isolated hepatic steatosis, which involves fat accumulation in the liver without accompanying inflammation or hepatocellular injury [1]. Indeed, given its link with obesity, the accumulation of fatty acids in the liver, subsequent increases in hepatic inflammation, and associated insulin resistance, SLD is generally viewed as the hepatic component of the metabolic syndrome [1,2]. The diagnosis of the pathological form of the disease, known as metabolic dysfunction-associated steatohepatitis (MASH), is defined as the presence of more than 5% hepatic fat and hepatic injury/ballooning [3]. An additional hallmark of this progression to MASH, from MASLD, is the presence of tissue scarring, with liver fibrosis being the main determinant of mortality [4]. Coupled with hepatic ballooning and inflammation, the resultant pathology can lead to irreversible cirrhosis.

It is now well established that low levels of serum albumin are predictive of hepatic-related mortality and morbidity in patients with MASLD [5,6,7,8,9,10,11], leading to the suggestion that albumin could be an early biomarker for diseases of liver disfunction [12]. Already, albumin is used as an indicator for advanced disease, with low levels a determining factor to perform a diagnostic liver biopsy [13,14]. Furthermore, human albumin has anti-inflammatory and antioxidant properties [15,16,17], the recognition of which has led to the initiation of a clinical trial to investigate changes to these markers in sepsis patients following administration of albumin (NCT03950778). In addition to this, plasma albumin levels have been shown to inversely correlate with weight, and has been suggested as an indicator for the development of type 2 diabetes [18,19]. Lastly, albumin is used as an intravenous treatment to extend plasma in hepatorenal syndrome, which can be a vascular-related complication of cirrhosis [20,21], making this already approved therapeutic ideal to be repurposed for treatment of other diseases. To our knowledge, therapeutic administration of albumin for MASLD has not been investigated, despite its inverse association with disease severity, and its well-established anti-inflammatory and antioxidant properties. Therefore, we questioned whether raising serum albumin levels through parenteral administration could have a therapeutic effect on MASLD and potentially prevent its progression to MASH. To achieve this, male C57BL/6 mice were placed on a diet of either standard chow (Std Chow) or a high-fat and cholesterol diet (PC) before being receiving intraperitoneal (i.p.) bovine serum albumin (BSA). Weight, metabolic function, hepatic steatosis and inflammation were then assessed. We showed that administration of BSA, dramatically prevented weight gain in a murine model of MASLD, and reduced hepatic inflammation.

## 2. Results

### 2.1. BSA Treatment Prevented Diet-Induced Weight Gain, However Had No Effect on Glucose Homestasis

**Figure 1:**
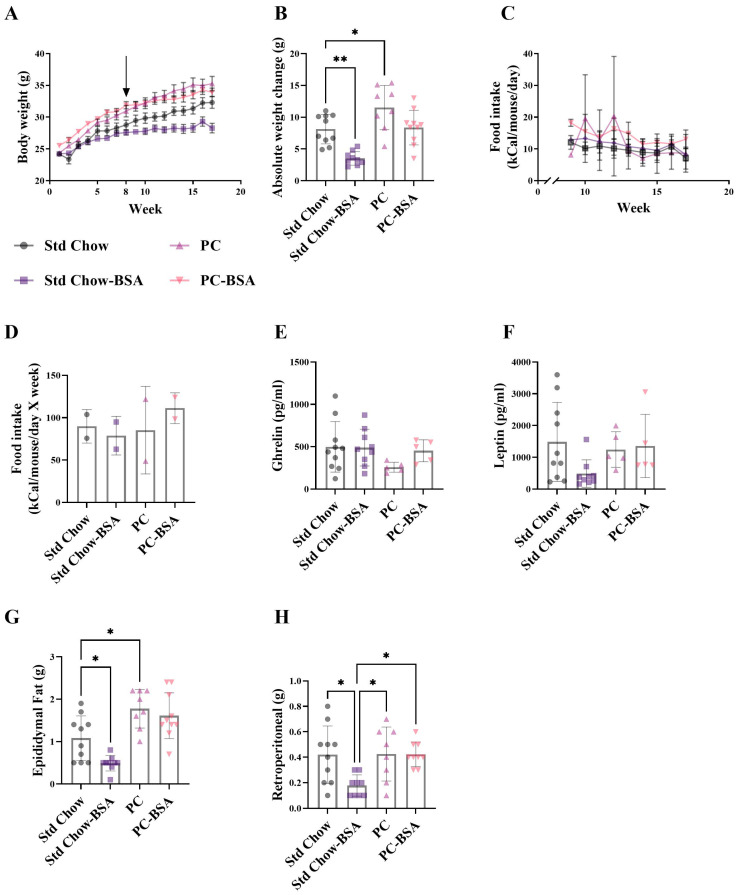
Body weight and visceral fat mass over 16 weeks. Mice were fed either a standard chow or high-fat, high-palmitate, and high-cholesterol diet for 8 weeks. At this timepoint, a subset of mice was treated with intraperitoneal injections of bovine serum albumin (BSA) delivered every three days for another 8 weeks (as indicated by arrow in (**A**)). (**A**–**D**) Mice were weighed weekly and their food intake was monitored. Plasma (**E**) ghrelin and (**F**) leptin concentrations were measured and (**G**,**H**) fat pads were weighed at the end of the 16 weeks. Results are mean ± standard deviation (SD) (*n* = 5–10), with significant differences determined by one-way ANOVA with Tukey’s post hoc analysis for normally distributed data; non-parametric one-way ANOVA with Dunn’s post hoc analysis for non-normally distributed data. * *p* < 0.05; ** *p* < 0.01. (**C**,**D**) Food intake measured for whole cages for 2 independent animal cages.

PC fed mice had a 1.5-fold increase in body weight compared to Std chow fed mice (Figure 1A,B; *p* = 0.0356), with no difference in food intake observed between the groups (Figure 1C,D). Interestingly, mice fed the PC diet showed slightly reduced plasma ghrelin concentrations, despite gaining significantly more weight, as compared to the standard chow-fed mice (Figure 1E), but showed no differences in leptin concentrations (Figure 1F). Treatment of the PC-fed mice with BSA resulted in 27% less weight gain (Figure 1B; *p* = 0.0575) resulting in body weights equivalent to that observed in the non-treated Std Chow fed mice. However, there was no difference in ghrelin or leptin concentration, nor the weight of either epididymal or retroperitoneal fat after BSA treatment compared to the tissue harvested from mice fed the PC diet (Figure 1G,H). Similarly, mice fed the Std Chow and treated with BSA gained 56.6% less weight than their non-treated counterparts (Figure 1B; *p* = 0.0019). In contrast to the mice fed the PC diet, this decrease in body weight was associated with slightly reduced plasma leptin (Figure 1F), and significantly less epididymal (Figure 1G; *p* = 0.0380) and visceral fat (Figure 1H; *p* = 0.0375) compared to the untreated mice.

**Figure 2:**
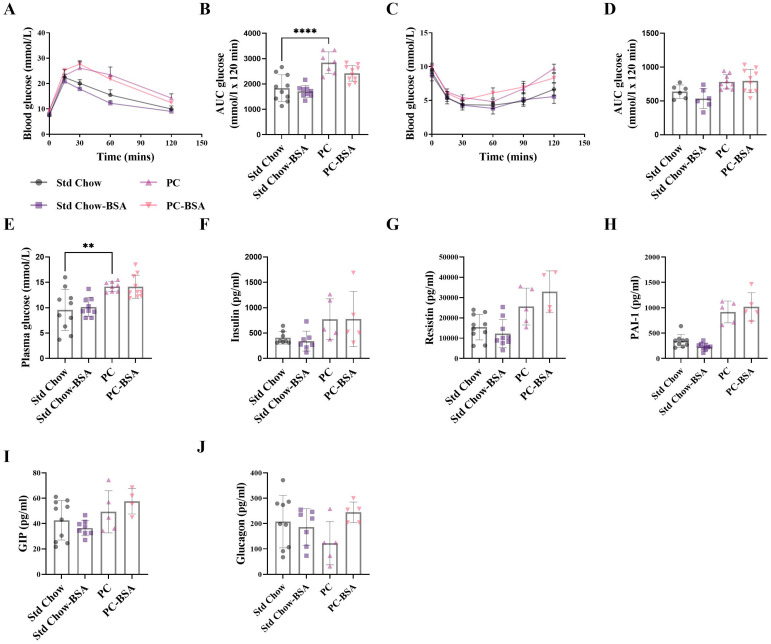
BSA treatment has no effect on metabolic outcomes. (**A**) A glucose tolerance test (GTT) was performed on 5 h fasted mice, where blood glucose was measured over 2 h and the (**B**) area under the curve (AUC) was calculated (*n* = 7–10). Similarly, (**C**) an insulin tolerance test (ITT) was performed, and the (**D**) AUC was calculated (*n* = 6–10). (**E**) Fasting blood glucose concentrations were measured from whole blood (*n* = 10), and (**F**) insulin (**G**) resistin, (**H**) plasminogen activator inhibitor-1 (PAI-1), (**I**) gastric inhibitory polypeptide (GIP) and (**J**) glucagon (*n* = 5–10) concentrations were measured from fasting plasma collected at the experimental endpoint. Results are mean ± SD, with significant differences determined by one-way ANOVA, with Tukey’s post hoc analysis used for normally distributed data; non-parametric one-way ANOVA, with Dunn’s post hoc used for non-normally distributed data. ** *p* < 0.01; **** *p* < 0.0001.

As weight-gain is commonly associated with perturbed glucose metabolism and insulin resistance (IR), leading to MASLD, we next investigated if the profound weight reduction recorded in the BSA treated mice translated to an improved metabolic status. The development of IR as a consequence of high-fat-diet-induced obesity was confirmed by glucose tolerance test (GTT), where predictably, the mice on the PC diet showed a significant impairment in glucose tolerance compared to Std chow-fed mice (Figure 2A,B; *p* < 0.0001). Despite the large differences in gross weight, mice treated with BSA showed no difference in their intolerance to glucose compared to mice on the PC diet (*p* = 0.1514). Although slightly increased, PC-fed mice did not develop IR as determined by insulin tolerance test (ITT), and consistent with the results for the GTT, treatment with BSA did not change the sensitivity to insulin (Figure 2C,D). To further determine any differences in glucose metabolism at the experimental endpoint, plasma was collected, and fasting levels of glucose and insulin were measured. Hyperglycaemia without hyperinsulinemia was evident in the PC-fed mice as compared to the Std chow-fed mice (Figure 2E,F; *p* = 0.0043 and *p* = 0.2208, respectively). Consistent with the ITT and GTT assays, treatment with BSA had no significant effect on either of these measures of metabolism. Further, the effect of BSA treatment on other hormones involved in glucose metabolism was assessed.

The concentration of plasminogen activator inhibitor 1 (PAI-1; *p* = 0.1004) was increased as a result of the PC diet, as expected, with no effect of BSA treatment (Figure 2H). No changes in concentrations of resistin, (Figure 2G), gastric inhibitory polypeptide (GIP; Figure 2I), or glucagon (Figure 2J) were seen for either diet or BSA treatment.

### 2.2. BSA Treatment Reversed Hepatic Fat Accumulation

**Figure 3:**
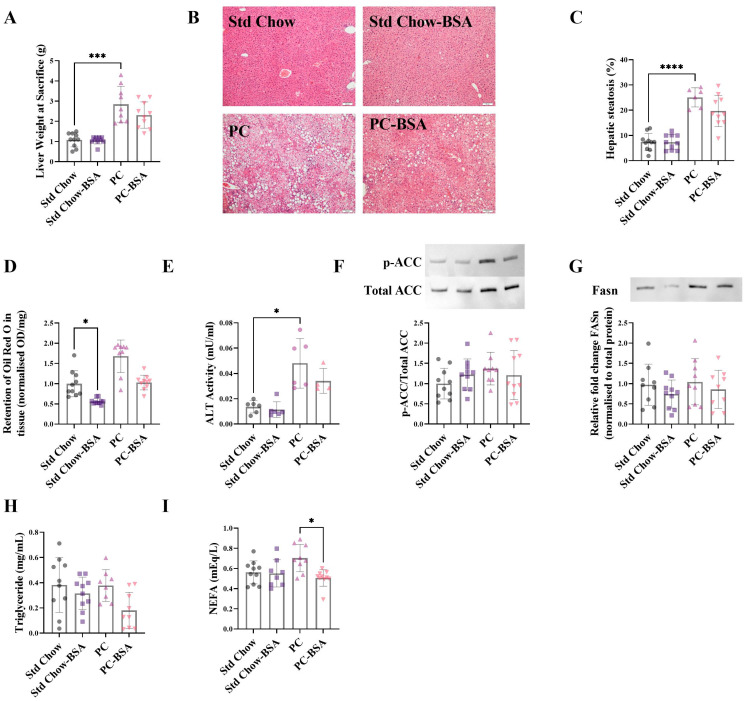
BSA treatment reduces hepatic fat accumulation. (**A**) Livers were weighed at the experimental endpoint. (**B**) Fixed liver samples were stained with haematoxylin and eosin. (**C**) Vacuoles observed on the histological sections indicate lipid droplets and were quantified to show the percentage steatosis (*n* = 6–10). (**D**) Frozen liver samples were homogenised and stained for neutral lipids with Oil Red O (*n* = 9–10). Data points are presented as optical density (OD) per mg of tissue, then normalised to Std Chow. (**E**) Plasma collected at the experimental endpoint was assayed for alanine aminotransferase (ALT) activity (*n* = 4–7). Total protein was extracted from frozen livers at the experimental endpoint and expression levels were assessed using Western blot (*n* = 8–10). (**F**) Acetyl-coA carboxylase (ACC); (**G**) fatty acid synthetase (FASn). ACC and FASn normalised to total protein; representative protein band shown. Plasma collected at the experimental endpoint was assayed for (**H**) triglyceride (*n* = 8–10) and (**I**) non-esterified fatty acids (NEFA) levels (*n* = 8–10). Results are mean ± SD, with significant differences determined by one-way ANOVA with Tukey’s post hoc analysis for normally distributed data; non-parametric one-way ANOVA with Dunn’s post hoc for non-normally distributed data. Magnification ×10, scale bar—50 µm. * *p* < 0.05; *** *p* < 0.001; **** *p* < 0.0001.

Correlating with the changes in glucose metabolism (Figure 2), mice on the PC diet showed a significant increase in liver weight as compared to the mice fed the Std Chow (Figure 3A; *p* = 0.0006). Histological analysis using haematoxylin and eosin (H&E) staining revealed hepatocyte ballooning in PC diet mouse livers with a 3.4-fold increase in the deposition of fat (Figure 3B,C; *p* < 0.0001). This finding is supported by quantitative lipid Oil Red O assay that showed a 1.7-fold increase in neutral lipid (triglyceride plus cholesterol esters) accumulation in the livers of the PC-fed mice compared to the livers from mice on the Std Chow (Figure 3D; *p* = 0.0878). Although the treatment of PC-fed mice with BSA reduced the percentage of steatosis in liver tissue, this did not reach significance (Figure 3C; *p* = 0.2716). However, the administration of BSA did significantly reduce the lipid accumulation in the Std Chow diet mouse livers (Figure 3D; *p*= 0.0321).

Alanine aminotransferase (ALT) is an enzyme released by the liver in response to liver damage, and is upregulated in MASLD [22]. A clear increase in ALT activity was shown in the mice on the PC diet compared to that of mice on the Std Chow diet (Figure 3E; *p* = 0.015). Although the BSA treatment decreased ALT activity in both diets compared to their respective controls, significance was not reached.

To investigate the mechanisms by which BSA treatment might be altering the levels of fats and lipids accumulating in the liver tissue, the protein levels of key enzymes involved in de novo lipogenesis were quantified. Fatty acid synthetase (FASn) catalyses the conversion of malonyl-CoA to palmitoyl-CoA. Upstream of this reaction, acetyl-CoA carboxylase (ACC), in its unphosphorylated state, catalyses the conversion of acetyl-CoA to malonyl Co-A, in what is often referred to as the rate limiting step in de novo lipogenesis. The PC diet and subsequent BSA treatment, had no effect on the levels of ACC or FASn (Figure 3F,G).

In addition to de novo synthesis of lipids within the liver, circulating lipids also contribute to the accumulation of fat in the liver. Accordingly, circulating non-esterified fatty acids (NEFA) and triglycerides were measured in the plasma of all mice at the experimental endpoint. Although there was no significant difference in the levels of plasma triglyceride between any treatment or diet group (Figure 3H), there was an increase in plasma NEFAs as a result of the PC diet, which was significantly reduced by BSA treatment (Figure 3I; *p* = 0.0184).

### 2.3. BSA Treatment Decreased Hepatic Inflammation

**Figure 4:**
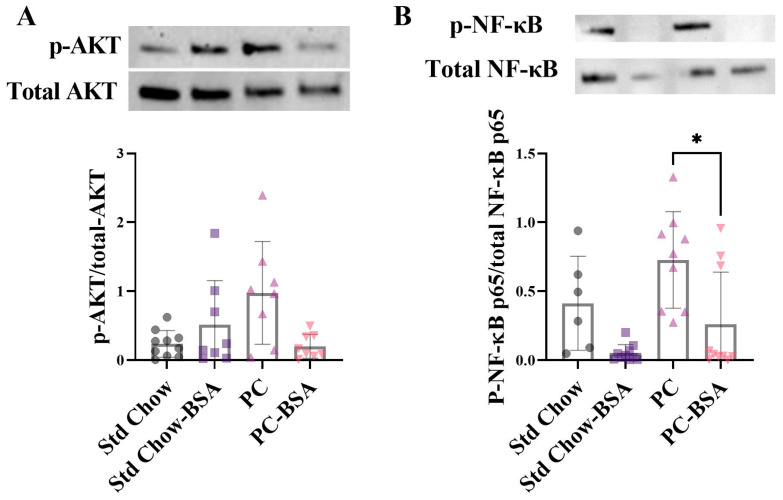
BSA treatment effect on measures of inflammation. Protein expression levels of (**A**) protein kinase B (AKT) and (**B**) nuclear factor-κB (NF-κB) were assessed by Western blot analysis from frozen liver samples (*n* = 6–10). Results are mean ± SD, with significant differences determined by non-parametric one-way ANOVA with Dunn’s post hoc for non-normally distributed data. * *p* < 0.05.

In MASLD, hepatic steatosis is present without evidence of inflammation, whereas when progressed to MASH, hepatic steatosis is associated with lobular inflammation and apoptosis that contributes to fibrosis and cirrhosis. Protein kinase B (AKT) has been shown to activate the NF-κB pathway via mammalian target of rapamycin (mTOR) in inflammatory disease models [23,24,25,26]. The transcription factor NF-κB is a key promoter of pro-inflammatory cytokine expression and is activated when phosphorylated. Thus, aligning with the expectation that prolonged steatosis results in hepatic inflammation, livers from mice fed the PC diet showed increased the levels of the phosphorylated AKT compared to tissue the mice fed the Std Chow diet (Figure 4A; *p* = 0.1976). Treatment of the PC-fed mice with BSA reduced this phosphorylation, although not significantly (*p* = 0.1528). Consistent with this, the phosphorylation of NF-κB was increased in the livers of mice on the PC diet compared to those of mice fed the Std Chow (Figure 4B). The treatment of mice with BSA significantly reduced this activation of NF-κB by 2.8-fold (*p* = 0.0328).

### 2.4. BSA Treatment of Mice Enhances the Expression of Genes Associated with Fibrotic Pathways

**Figure 5:**
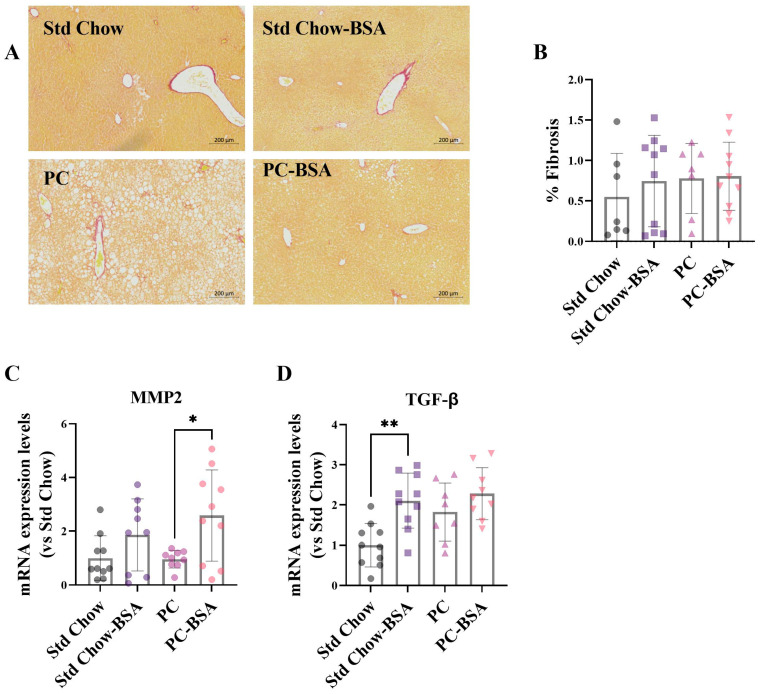
PC diet did not induce hepatic fibrosis. (**A**) Fixed livers were stained with Picro Sirius Red for fibrosis, and (**B**) the percentage fibrotic area was calculated (*n* = 8–10). Total RNA was extracted from liver samples and mRNA gene expression levels were assessed using RT-qPCR (*n* = 8–10). (**C**) Transforming growth factor-β (TGF-β); (**D**) Matrix metalloproteinase-2 (MMP2). Results are mean ± SD, with significant differences determined by one-way ANOVA with Tukey’s post hoc analysis for normally distributed data. Magnification ×10, scale bar—200 µm. * *p* < 0.05; ** *p* < 0.01.

The combination of increased steatosis and inflammation in the liver causes MASLD to progress to MASH, the more advanced form of disease, which presents with liver fibrosis. The combined reduction in steatosis and liver inflammation as a result of the BSA treatment suggested that the transition from MASLD to MASH may be prevented. However, despite an expectation that the PC diet would result in pathology characteristic of MASH with fibrosis, staining sections of liver tissue with Picro Sirius Red showed no significant evidence of fibrotic change in the livers of any mice (Figure 5A,B). To explore this further, the expression levels of two genes that are involved in the biological pathways leading to fibrosis were measured. Transforming growth factor-β (TGF-β) is an initiator of fibrosis, production of which correlates to fibrosis progression, promoting downstream secretion of extracellular matrix (ECM) proteins [27]. Matrix metalloproteinases (MMP)-2 has anti-fibrotic effects, as MMP2^−/−^ mice show increased type 1 collagen expression, although the mechanism by which this happens is unclear [28].

While the PC diet had no effect on MMP2 expression (Figure 5C), it did lead to an increased expression of TGF-β mRNA in liver tissue compared to Std Chow-fed mice (Figure 5D), although significance was not reached (*p* = 0.0540). Treatment of PC-fed mice with BSA resulted in a slight increase in TGF-β gene expression, and a 2.7-fold increase in MMP2 gene expression (Figure 5C; *p* = 0.0249). BSA treatment of Std Chow-fed mice also resulted in a significant increase in TGF-β gene expression (Figure 5D; *p* = 0.0030), and a modest increase in MMP2 (Figure 5C).

## 3. Discussion

Despite being the leading cause of liver disease and with a global prevalence of approximately 30%, and 10-year projections suggesting this could rise to more than 55%, there remains no specific pharmacological intervention for treatment of MASLD, formerly non-alcoholic fatty liver disease [29,30]. Further, approximately one third of patients with MASLD progress to MASH, although the true value is largely unknown due to the fact that for those patients who voluntarily undergo liver biopsies to diagnose MASH, MASH is already suspected [31]. Therefore, it is imperative to find a pharmacological solution for when non-pharmacological treatments fail to prevent pathological disease progression and reverse MASLD. Weight loss is the first-line treatment recommended for MASLD [13,14]. Even short-term weight loss has been shown to decrease inflammatory markers and waist circumference [32,33], both of which contribute to the development of insulin resistance. However, it has been reported that less than 50% of people are successful in achieving the necessary loss of ≥7% body weight required for a beneficial effect on liver health [34]. For these patients it has been suggested that they are offered anti-obesity medications as adjunct therapy to lifestyle changes to reduce the risk of MASLD-related disease progression [35]. There are currently seven FDA-approved weight loss prescription medications; however, all have unpleasant gastrointestinal side effects, as well as other side effects ranging from mild dry-mouth to the severe depression and suicidal ideation [35,36]. In contrast to this, adverse reactions to intravenous albumin are generally mild and transient, and usually disappear when infusion is stopped [37,38,39]. Reduced plasma albumin has been associated with increased body weight and impaired glucose regulation [18,19]. Additionally, low plasma albumin correlated with prediction of development of type 2 diabetes [19], while not diagnostic, can be an indicator of advanced disease progression [13,14] and is already clinically approved, suggesting warranted investigation to determine whether restoring albumin levels by could prevent disease progression. Therefore, we sought to determine whether parenteral administration of albumin would confer protection against weight gain and glucose impairment induced by a high fat/high cholesterol diet. Accordingly, to our knowledge, this is the first study to demonstrate the anti-obesity effect of BSA and to show that BSA treatment reduced hepatic fat accumulation to improve liver function.

In this study, we showed a remarkable reduction in weight gain of mice receiving BSA irrespective of whether they were fed Std Chow or the high fat/high cholesterol diet. However, only mice fed the Std Chow and treated with BSA showed a correlating weight reduction in the mass of the visceral fat pads. One possibility for this observation is that weight loss occurs in specific fat depositories first. In addition to the two fat pads measured here, the subcutaneous fat, as well as the weight of fat surrounding internal organs, contribute to overall body weight. Given that BSA-treated mice fed Std Chow gained less gross body weight which, reflected as reduced epididymal and retroperitoneal fat weight, while BSA-treated mice fed the PC diet also gained less gross body weight, but with reduced liver weight instead of fat loss in those depots, this suggests that BSA treatment may initially target alternative fat depots, as well as the subcutaneous fat depots, for weight loss. This possibility was supported by analysis of the liver where gross liver weight reduction in BSA treated PC-fed mice showed concurrent reduction in steatosis, whereas there was no difference in gross liver weight or steatosis between BSA treated and untreated Std Chow-fed mice. Mechanistically, this finding suggests that as a supplement, albumin may enhance weight loss. While there is some debate [40], it has been acknowledged that protein enriched diets can lead to greater improvements in weight loss [41,42]; however, it is unknown whether albumin specifically contributes. The mechanism behind protein-induced weight loss has been proposed to be simply a result of satiation from the protein, and therefore weight loss occurs due to reduced caloric intake [43]. Research into the specific effect of plasma albumin on food intake found that reduced albumin levels were associated with increased food intake [18]. Recording the weight difference in food hoppers over 24 h showed no significant changes in food intake, although significant wastage of food was also recorded where the animals pulled the food out of the hopper, but did not consume it. Regardless, a slight decrease in food consumption by BSA-treated Std chow-fed mice, compared to the untreated control, suggests that there might be the inverse effect, where supplemented albumin reduces food intake. Interestingly, leptin, a hormone which is known to suppress appetite, was also reduced only in Std chow-fed BSA treated mice suggesting that reduced food consumption could, in part, explain the reduced weight of this group. Further investigation will be required to determine the mechanism by which BSA is affecting weight loss.

In addition to the simple deposition of fat in organs as a result of increased dietary intake, the accumulation of lipids in the liver is enhanced by activation of the de novo lipogenesis pathway. However, the observation that neither the expression of FASn or the phosphorylation of ACC were increased in the liver tissue from the PC-fed mice indicated that the hepatic steatosis in this murine model was not occurring through the de novo lipogenesis pathway. While unexpected, it has been reported that de novo lipogenesis is responsible for only 26% of hepatic triglyceride in MASLD patients. Instead, the plasma NEFA pool, emanating from the adipose tissue, contributes 60% of the triglyceride content of the liver of MASLD patients [44]. As adipose becomes insulin resistant, it does not respond to the lipolysis-suppressive effects of insulin, raising the levels of circulating NEFA, which can then accumulate in the liver. The administration of BSA to mice reduced the hyperinsulinemia caused by the PC diet, which conceivably increased the insulin sensitivity of the adipose tissue, normalising lipolysis and thus reducing circulating NEFA, translating to reduced triglyceride accumulation in the liver.

The accumulation of fat in the liver and insulin resistance contribute to the initiation of pro-inflammatory immune responses, which results in the development of fibrosis, a hallmark feature of MASH, that can then subsequently progress to cirrhosis [45]. In this study, the predominant transcriptional activator of pro-inflammatory cytokines (NF-κB) was shown to be activated in the liver of PC-fed mice. Treatment with BSA resulted in the downregulation of NF-κB activation indicating a decrease in hepatic inflammation. Albumin is a known antioxidant with immunomodulatory properties which are regularly clinically relied upon for the treatment of cirrhosis [46,47,48,49,50]. Albumin modulates oxidative stress by increasing glutathione, thereby reducing TNF-mediated NF-κB activation [15,51,52]. In patients with decompensated cirrhosis, treatment with human serum albumin was shown to reduce the degree of systemic inflammation via the inhibition of the NF-κB pathway [53,54].

Moreover, our findings align with the role of albumin in hepatic protection, as demonstrated by its ability to safeguard the liver from TNF-induced injury, much like its demonstrated capacity to reduce LPS-mediated production of TNF following preconditioning of mice with albumin [54,55,56]. Furthermore, albumin administration has been shown to reduce inflammation and oxidative stress in models of high-fat feeding and acute injury [57,58]. One recently proposed explanation for this is circulating fatty acid and its effect on albumin oxidation capacity. Uzelac et al. recently showed that increasing the ratio of albumin to fatty acids had a negative effect on antioxidant activity, and suggested that this may contribute to the increased oxidative stress in conditions where fatty acids and glucose are increased, such as in type 2 diabetes [59].

Given the reduction in steatosis and inflammation induced by BSA treatment, it was expected that this would translate to the prevention of the progression to fibrosis. Unfortunately, the PC diet did not induce fibrosis; however, did it result in an upregulation of TGF-β, a gene that is instrumental in the induction of ECM [27]. The further enhancement of TGF-β expression after treatment with BSA in not only the PC-fed, but also the Std Chow-fed mice was surprising. However, independently of its role in the initiation of fibrosis, TGF-β has also been shown to have anti-inflammatory and anti-fibrotic functions where TGF-β secreted from CD4+ T regulatory cells induced IL-10 secretion which in turn regulated TGF-β-mediated fibrosis [60]. Further, MMP2, a type IV collagenase typically associated with fibrosis, was also increased in both BSA treatment groups. However, independently of its role in the initiation of fibrosis, TGF-β has also been shown to have anti-inflammatory and anti-fibrotic functions where TGF-β secreted from CD4+ T regulatory cells induced IL-10 secretion which in turn regulated TGF-β-mediated fibrosis [60]. Further, MMP2, a type IV collagenase typically associated with fibrosis, was also increased in both BSA treatment groups. While most MMPs disrupt basement membranes and allow for inflammatory cells to be easily recruited to the site of injury, MMP2 can also suppress the expression of collagen 1 and is thus considered to be anti-fibrotic. Indeed, it has been demonstrated that in MMP2^−/−^ mice, fibrosis was doubled compared to control mice [61]. Based on these observations, it can be postulated that the effect of BSA on TGF-β observed in our study may be a negative feedback loop necessary for the protection of the liver through activation of the anti-fibrotic properties of MMP2.

## 4. Materials and Methods

### 4.1. Animal Husbandry

Seven-week-old C57BL/6 male mice (*n* = 40) were purchased from Australian Bioresources (Sydney, Australia) and housed in a 12 h light/dark cycle with the room temperature 22 ± 2 °C in filtered Greenline GM500 cages containing Alpha-Dri^®^ bedding (Shepherd Specialty Papers, Milford, NJ, USA). To ensure optimal animal welfare, a maximum of 5 mice, and a minimum of 2, were housed per cage, and environmental enrichment in the form of nesting materials, cardboard tubes, wooden climbing blocks, and mouse houses. All animal procedures were approved by the University of Technology Animal Care and Ethics Committee (Ethics approval number: UTS ACEC ETH18-2248.

### 4.2. Animal Model of Metabolic Dysfunction-Associated Steatotic Liver Disease

**Figure 6:**
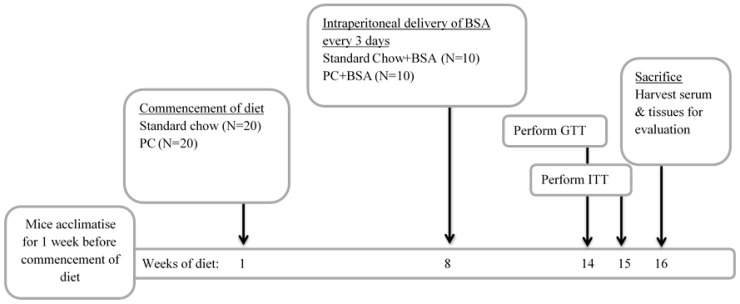
Experimental design of metabolic dysfunction-associated steatotic liver disease (MASLD) model. Mice were fed a diet of either standard chow or diet to induced MASLD (PC diet) commencing in week 1 of the diet. Treatment of mice began in the 8th week of the diet. In weeks 14 and 15 of the diet, an intraperitoneal GTT and intraperitoneal ITT were performed, respectively. Animals were euthanized and tissue harvested in the 16th week of the diet.

Mice were allowed to acclimate for 1 week before they were randomised into two groups (*n* = 20) and transitioned to ad libitum feeding on either standard chow (Std Chow; 68.3% carbohydrate, 4.1% fat; TD.94048, Biological Associates, Sydney, Australia) or a diet that is high in fat to induce MASLD (Palmitic acid-cholesterol diet [PC]; 46.2% carbohydrate, 29.0% fat; TD.160785, Biological Associates, Sydney, Australia). After 8 weeks of feeding, a subset of mice (*n* = 10) from each group received bovine serum albumin (BSA; Sigma Aldrich, Sydney, Australia) dissolved in saline (0.8 mg/kg) via intraperitoneal (i.p.) injection every three days for the next 8 weeks of their diets (Figure 6). At weeks 14 and 15, intraperitoneal GTT and ITT were performed, respectively. This experimental time frame was chosen as male C57BL/6 mice have been shown to have increased fat mass and adiposity following 8 weeks of high fat diet feeding [62]. Further, by 15 weeks high fat diet feeding, mice exhibited impaired basal insulin and glucose tolerance, compared to only 3 weeks on a high fat diet [63]. At the experimental endpoint (16 weeks on diets in total), the animals were fasted for 5 h before they were euthanised by cardiac puncture after full anaesthesia has been achieved using isoflurane. Liver and visceral fat pads were weighed, and portions of tissue were fixed in 4% paraformaldehyde or snap frozen in liquid nitrogen for downstream analysis. Whole blood was collected in heparinised syringes and centrifuged (13,000× *g* for 5 min) for the collection of plasma, which was stored at −20 °C.

### 4.3. Measurement of the Metabolic Status of Animals

To determine the effect of the diet and the BSA treatment on glucose metabolism, i.p. GTTs and ITTs were performed. Briefly, for the GTT, mice were fasted for 5 h before basal blood glucose readings were taken using a Accu-Check Performa (Roche Diagnostics, Indianapolis, IN, USA). A bolus of glucose was then administered via i.p. injection (2g D-glucose/kg body weight) before measurement of the blood glucose at 15, 30, 60 and 120 min post injection. Similarly, for the ITT, NovoRapid^®^ insulin (Novo Nordisk Pharmaceuticals Pty Ltd., Sydney, Australia) was delivered via i.p. injection (0.001 IU/g mouse) and blood glucose levels monitored over 120 min. The area under the curve (AUC) of glucose concentration was calculated for each mouse. Fasting insulin, resistin, gastric inhibitory polypeptide (GIP), plasminogen activator inhibitor-1 (PAI-1), glucagon, ghrelin and leptin concentration in plasma were measured using a commercial multiplex assay system (Bio-Plex ProTM Mouse diabetes panel, Bio-Rad Laboratories, Hercules, CA, USA) according to the manufacturer’s instructions.

### 4.4. Biochemical Analysis of Plasma

Alanine aminotransferase activity was measured in collected plasma using a commercially available assay kit (BioVision Inc., Milpitas, CA, USA) and non-esterified fatty acids (NEFA) were quantified in the plasma using the LabAssay^TM^ NEFA assay kit (FUJIFILM Wako Chemicals USA Corporation, Richmond, VA, USA).

Intrahepatic neutral lipid accumulation assay Intrahepatic neutral lipid was assessed as previously described [64]. Briefly, 80–100 mg of liver tissue was homogenised in 1 mL of Oil Red O working solution (1:1.5 Oil Red O stock, [ProSciTech, Townsville, QLD, Australia]: 1% dextrin [Sigma Aldrich, Sydney, Australia]) with 1.4 mm Zirconium oxide beads (Precellys 24, Montigny-le-Bretonneux, France) using a Minilys (Bertin Technologies, Montigny-le-Bretonneux, France). Samples were then vortexed for 15 min to ensure even distribution of stain through the tissue, then centrifuged at 12,300× *g* for 2 min, and the supernatant removed. Oil Red O stain was extracted using 500 µL 99% isopropanol and 100 µL of each sample was transferred to a 96 well plate and the absorbance read at 520 nm. The fold change in Oil Red O staining per gram of tissue normalised to the Std Chow was then calculated.

### 4.5. Histological Examination of Liver Tissue

Fixed tissue samples were processed and embedded in paraffin. Paraffin embedded sections were cut at 5 μm, were deparaffinized, then were rehydrated and routine H&E staining was carried out for the determination of hepatic steatosis [65]. Picro Sirius red staining was used for the determination of fibrosis, where collagen depositions are stained red [66]. Briefly, rehydrated sections were incubated in the Picro Sirius Red stain (Abcam, Cambridge, UK) at room temperature for 60 min. Slides were then washed with the supplied acetic acid solution twice, followed by a wash in 100% ethanol. Slides were then dehydrated, mounted and cover slipped.

For quantification, slides were visualised on a BX51 Upright Fluorescence Microscope, and three field of views of each section, with three sections per animal (nine images total, per animal), were imaged at 10× magnification. Percentage fat and percentage fibrosis were calculated using the threshold function on Fiji (version 6.4) [67].

### 4.6. Western Blot Analysis

Whole protein lysate was extracted from 30 to 40 mg of frozen livers using RIPA lysis buffer (1% IGEPAL, 0.1% SDS, 0.5% sodium deoxycholate, 150 mM NaCl, 50 mM Tris, pH 8). Total protein samples (15 µg) were resolved on a 4–15% Mini Protean TGX stain free protein gel (Bio-Rad Laboratories, Hercules, CA, USA), then transferred to a PVDF membrane, and blocked with skim milk (5% *v*/*v* in 1X TBST) for 2 h at room temperature. Blots were then incubated with primary antibodies (Supplementary Table S1) overnight at 4 °C. After washing with TBST (3X), the membranes were incubated with the relevant secondary antibody (Supplementary Table S1) conjugated to HRP for 2 h at room temperature. Proteins were then visualised using ECL substrate (Bio-Rad Laboratories, Hercules, CA, USA) and a Bio-Rad Chemidoc Imaging System^TM^. Band intensity was quantified using Fiji or normalised to the total protein using the Bio-Rad Image Lab software 6.0.

### 4.7. Quantification of Gene Expression Using RT-qPCR

Total RNA was extracted from 40 mg of frozen tissues using TriSure reagent (Meridian Bioscience, Bioline Reagents, Inc., Taunton, MA, USA). RNA (800 ng/µL) was then reverse transcribed using the Tetro cDNA Synthesis Kit (Meridian Bioscience, Bioline Reagents, Inc., Taunton, MA, USA) and Bio-Rad T100^TM^ Thermocycler. Gene expression levels (primer sequences are in Supplementary Table S2) were then quantified using SYBR green (Meridian Bioscience, Bioline Reagents, Inc., Taunton, MA, USA) using the Bio-Rad CFX96 thermocycler. Relative changes in mRNA levels were determined by the 2^ΔΔCT^ method [68], using β-actin as the reference gene.

### 4.8. Statistical Analysis

All results are expressed as mean ± SD. A Shapiro–Wilk test was performed to determine normal distribution of data. One-way ANOVA was applied to normally distributed data with Tukey’s post hoc tests used to determine significance of the results. Non-parametric ANOVA (Kruskal–Wallis test) was applied to non-normally distributed data with Dunn’s multiple comparisons post hoc used to determine significance of the results. Statistical analysis and graphical presentation performed using GraphPad PRISM version 9.1 (GraphPad Software, San Diego, CA, USA).

## 5. Conclusions

In summary, the data in this study determined that treatment with BSA prevents time-dependent weight gain, regardless of diet, and that BSA treatment can improve obesity-induced hyperglycaemia, reduce hepatic fat accumulation, and initiate an anti-fibrotic responses in the liver. Whilst further studies are still required to investigate the mechanism that mediates these beneficial effects, our findings provide new insights toward the development of albumin, either alone or in conjunction with other therapeutic approaches, to reduce hepatic steatosis. This study was limited the unexpected absence of fibrotic changes by the PC diet, and therefore, further investigation into the anti-fibrotic effects of parenteral BSA administration should be investigated.

From a clinical perspective, albumin presents an attractive treatment option, as it is already prescribed in the treatment of cardiovascular and renal complications from advanced liver disease, as well as sepsis [20]. These treatments are given via intravenous infusion. In this study, i.p. injection was chosen for the delivery of albumin due to convenience from a laboratory perspective. Whether oral or intravenous delivery is also efficacious in reducing total body weight will need to be investigated in future studies.

## Data Availability

All relevant data are contained within this article.

## Supplementary Materials

The following supporting information can be downloaded at: https://www.mdpi.com/article/10.3390/ijms26157156/s1.
