# Supplementary material for: Albumin Reduces Hepatic Steatosis and Inflammation in High-Fat-Diet-Fed Mice
Source: Int J Mol Sci. 2025 Jul 24;26(15):7156. doi: 10.3390/ijms26157156 (PMC12346722; doi:10.3390/ijms26157156)
Supplement: Supplementary file 1 [file ijms-26-07156-s001.zip › ijms-3747835-supplementary.pdf]

Supporting Information: Albumin reduces hepatic steatosis and inflammation in high fat diet-fed mice

Claire Rennie[1], Sheila Donnelly[1,2], Kristine C. McGrath[1*]

[1]School of Life Sciences, University of Technology Sydney, Sydney, Australia

[2]School of Natural Sciences, University of Galway, Galway, Ireland

MATERIALS AND METHODS: SUPPLEMENTARY TABLES

Table S1: Antibodies used in Western blot analysis

Protein	Dilution	Company	Catalogue number
β-Actin	1:2500	Abcam	8226
GAPDH	1:2500	Cell Signalling Technology	5174T
NF-κB p65	1:5000	Cell Signalling Technology	3034
Phosphorylated NF-κB p65 (Ser536)	1:5000	Cell Signalling Technology	3033
AMP-activated protein kinase	1:2000	Cell Signalling Technology	2532
Phospho-AMPKα (Thr172)	1:500	Cell Signalling Technology	2535
Acetyl-CoA Carboxylase	1:2500	Cell Signalling Technology	676
Phospho-Acetyl-CoA Carboxylase (Ser79)	1:2500	Cell Signalling Technology	11818
Fatty Acid Synthetase (C20G5)	1:2000	Cell Signalling Technology	3180
Anti-rabbit secondary	1:10,000	Cell Signalling Technology	7074P2
Anti-mouse secondary	1:10,000	GE Health Care (now Cytiva)	NXA931-1ML

Table S2: Primer sequences used for qPCR

Gene	Forward primer	Reverse primer
β-Actin	5'- AGCCATGTACGTAGCCATCC	5'- CTCTCAGCTGTGGTGGTGAA
TNF	5'- CTATGTCTCAGCCTCTTCTC	5'- CATTTGGGAACTTCTCATCC
MMP2	5'-CAGGGCACCTCCTACAACAG	5'-CAGTGGACATAGCGGTCTCG
TGF-β	5'-GCTGAACCAAGGAGACGGAA	5'-ATGTCATGGATGGTGCCCAG
